# A topological transformation in evolutionary tree search methods based on maximum likelihood combining p-ECR and neighbor joining

**DOI:** 10.1186/1471-2105-9-S6-S4

**Published:** 2008-05-28

**Authors:** Mao-Zu Guo, Jian-Fu Li, Yang Liu

**Affiliations:** 1 Department of Computer Science and Technology, Harbin Institute of Technology, Harbin, 150001, P.R. China

## Abstract

**Background:**

Inference of evolutionary trees using the maximum likelihood principle is NP-hard. Therefore, all practical methods rely on heuristics. The topological transformations often used in heuristics are Nearest Neighbor Interchange (NNI), Subtree Prune and Regraft (SPR) and Tree Bisection and Reconnection (TBR). However, these topological transformations often fall easily into local optima, since there are not many trees accessible in one step from any given tree. Another more exhaustive topological transformation is p-Edge Contraction and Refinement (p-ECR). However, due to its high computation complexity, p-ECR has rarely been used in practice.

**Results:**

To make the p-ECR move more efficient, this paper proposes a new method named p-ECRNJ. The main idea of p-ECRNJ is to use neighbor joining (NJ) to refine the unresolved nodes produced in p-ECR.

**Conclusion:**

Experiments with real datasets show that p-ECRNJ can find better trees than the best known maximum likelihood methods so far and can efficiently improve local topological transforms in reasonable time.

## Background

The inference of evolutionary trees with computational methods has many important applications in medical and biological research, such as drug discovery and conservation biology. A rich variety of tree reconstruction methods based on sequences have been developed, which fall into three categories, (a) maximum parsimony methods, (b) distance based methods and (c) approaches applying the maximum likelihood principle. The latter two are the most popular. Distance based methods calculate pair-wise distances between the sequences with each other, and support the tree that best fits these observed distances. The prominent distance based method is Neighbor joining (NJ) [1], in which partial trees are iteratively combined to form a larger tree in a bottom-up manner. Due to low computational time complexity and demonstrated topological accuracy for small data sets, NJ and its variants have been widely used.

Maximum likelihood methods aim to find the tree that gains the maximum likelihood value to have produced the underlying data. A number of studies [2,3] have shown that maximum likelihood programs can recover the correct tree from simulated datasets more frequently than other methods, which supports numerous observations from real data and explains their popularity.

However, the main disadvantage of maximum likelihood methods is that they require much computational effort. Maximum likelihood reconstruction consists of two tasks. The first task involves edge length estimation: Given the topology of a tree, find edge lengths to maximize the likelihood function. This task is accomplished by iterative methods such as expectation maximization or using Newton-Raphson optimization. Each iteration of these methods requires computations that take on the order of the number of sequences times the number of sequence positions. The second, more challenging, task is to find a tree topology that maximizes the likelihood. The number of potential topologies grows exponentially with the number of sequences n, e.g. for n = 50 sequences there already exist 2.84*10^76 ^alternative topologies; a number almost as large as the number of atoms in the universe (≈10^80^). In fact, it has already been demonstrated that finding the optimal tree under the maximum likelihood criterion is NP-hard [4]. Consequently, the introduction of heuristics to reduce the search space in terms of potential topologies evaluated becomes inevitable, such as, the hill climbing based reconstruction algorithms [5-7]; the genetic algorithm based ones [8,9], etc.

Although using different search strategies, the heuristics are all to try to improve a starting tree/starting trees by a series of elementary topological rearrangements, until local optima is found. It is obvious that the performance of the heuristics depends on the degree of exhaustiveness of the topological rearrangements on some extent. The three often used topological rearrangements include Nearest Neighbor Interchange (NNI), Subtree Prune and Regraft (SPR) and Tree Bisection and Reconnection (TBR).

**Figure 1:**
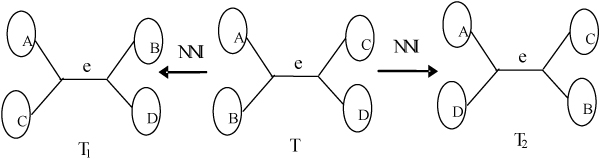
NNI.

The NNI move swaps one rooted subtree or leave on one side of an internal edge e with another on the other side. For every internal edge, a NNI move can produce two different topologies as shown in Figure 1. For a tree containing n sequences, the size of the neighborhood induced by NNI is *O*(*n*). The neighborhood of an evolutionary tree T under a topological rearrangement move is defined as be the set of all trees that can be obtained from T by one move.

A SPR move on a tree T is defined as cutting any edge and thereby pruning a subtree t, and then regrafting the subtree by the same cut edge to a new vertex obtained by subdividing a pre-existing edge in T-t. For a tree containing n sequences, the size of the neighborhood induced by SPR is O(n^2^).

**Figure 2:**
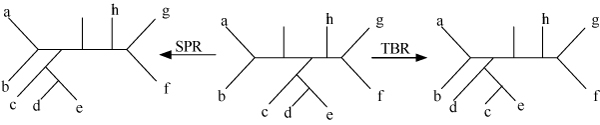
SPR and TBR.

In a TBR move an edge is removed from T, creating subtrees t and T-t, and then a new edge is added between the midpoints of any two edges in t and T-t, creating a new tree. For a tree containing n sequences, the size of the neighborhood induced by TBR is O(n^3^). See Figure 2 for schematic representation of SPR and TBR.

**Figure 3:**
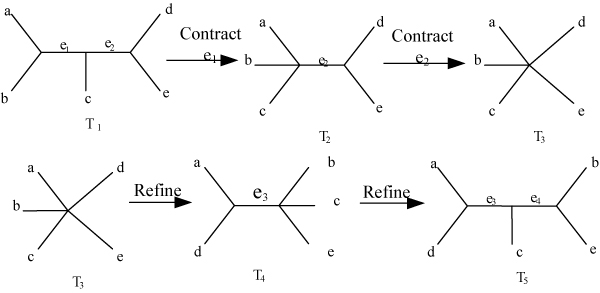
The illustration of a 2-ECR process.

As shown above, the neighborhood size produced by NNI, SPR and TBR acting on an evolutionary tree T comprising of n sequences is O(n), O(n^2^)and O(n^3^) respectively. Thus, TBR are the most exhaustive. Even TBR searches, however, can often get trapped in local optima, since there are not many trees accessible in one step from any given tree, which motivates the introduction of p-Edge Contraction and Refinement (p-ECR) [10]. A p-ECR move means to contract p edges all at once, creating unresolved nodes in the process, then refine these unresolved nodes to give back a binary tree. A contraction collapses an edge in the tree and identifies its two end points, while a refinement expands an unresolved node into two nodes connected by an edge. For example, the trees *T*_1 _and *T*_5 _in Figure 3 are separated by one 2-ECR move. From the definition of p-ECR, NNI is the special case of p-ECR when p equals to 1.

Let *S*_*u *_be the number of unresolved nodes produced in p-ECR and *d*_*i *_the degree of the unresolved node *i*, since the number of trees produced by n sequences is (2n-5)!!, refining the unresolved nodes can produce $\prod_{i=1}^{S_{u}}\left(\left(2d_{i}-5\right)!!\right)$ different trees. When p(>1) edges are deleted from a tree, the location relationship between the deleted edges determines the number of unresolved nodes produced and the degrees of the unresolved nodes. Now two extreme special cases are analyzed.

The first extreme special case is when all the p edges are adjacent. In this case, only one unresolved node with degree-(2p) is produced. Then the number of trees produced by one p-ECR is (4p-5)!!. Another extreme special case is when all the *p *edges disjoin. In this case, p unresolved nodes with degree-4 are produced. Then the number of trees produced by one p-ECR is ((2 × 4–5)!!)^p^, that is 3^p^.

In other cases, the number of possible trees produced is intermediate of the two special cases. Observe that there are $C_{n}^{p}$ ways of selecting p edges to contract, there are Ω(*n*^*n*^3^*p*^) trees produced by p-ECR. Thus, although an every sequence of p NNI moves on a tree is a p-ECR move on that tree, there are p-ECR moves that can not be performed by a sequence of p NNI move(the neighborhood size produced by p NNI moves is O(n^p^)). With such a wide search space, getting trapped in bad local optima can often be avoided, resulting in an exhaustive local search. Moreover, the exhaustiveness degree of a p-ECR move is dependent on the value of p, that is, a larger p means a larger search space for the correct tree, which could be potentially useful in selecting a suitable range of p.

However, how to quickly select the best one from so many possible evolutionary trees is a hard problem facing the p-ECR move, since there are so many potential topologies to evaluate and it is very time-consuming to compute the likelihood of a given topology as mentioned above. The straight answer is to simply evaluate every potential tree and select the best. Even for medium size of p, the answer is apparently impossible. Until now, there is no an efficient and general implementation of p-ECR. Consequently, people often yield up the exhaustive p-ECR and turn to some simpler one, such as NNI. In order to make p-ECR efficient, a method called p-ECRNJ motivated by NJ is presented in this paper. The main idea of p-ECRNJ is to use NJ to refine the unresolved nodes produced in p-ECR. In this paper, we use NJ to refine the unresolved nodes to improve p-ECR.

### NJ

NJ is a greedy algorithm, which attempts to minimize the sum of all branch-lengths on the constructed tree. Conceptually, it starts out with a star-formed tree where each leaf corresponds to a sequence, and iteratively picks two nodes adjacent to the root and joins them by inserting a new node between the root and the two selected nodes. When joining nodes, the method selects the pair of nodes i, j that minimizes

(1) *Q*_*ij *_= (*r *- 2)*d*_*ij *_- (*R*_*i *_+ *R*_*j*_)

where *d*_*ij *_is the distance between node i and j(assumed symmetric, i.e., *d*_*ij *_= *d*_*ji*_), *R*_*k *_is the sum over row *k *of the distance matrix: *R*_*k *_= ∑_*x*_*d*_*kx *_(where x ranges over all nodes adjacent to the root node), and r is the remaining number of nodes adjacent to the root. Once the pair i, j to agglomerate is selected, a new node *C *which represents the root of the new cluster is created. Then the length of branches (*C*, *i*) and (*C*, *j*) is estimated by the following Eq. (2)

(2) $$d_{Ci}=\frac{1}{2}\left(d_{ij}+\frac{R_{i}-R_{j}}{r-2}\right),d_{Cj}=\frac{1}{2}\left(d_{ij}+\frac{R_{j}-R_{i}}{r-2}\right)$$

Finally the distance matrix is reduced by replacing the distances relative to sequence *i *and sequence *j *by those between the new node C and any other node k using

(3) $$d_{Ck}=\frac{1}{2}\left(d_{ik}+d_{jk}-d_{ij}\right)$$

**Figure 4:**
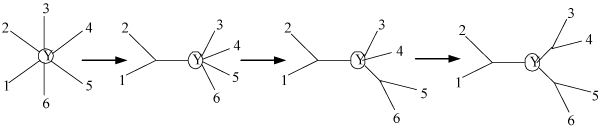
The illustration of NJ.

This formulation of NJ gives rise to a canonical algorithm that performs a search for min_*i*, *j*_*Q*_*ij*_, using time *O*(*r*^2^), and joins i and j, using time O(r) to update d. The search and joining is continued until only there three nodes are adjacent to the root. The total time complexity becomes *O*(*n*^3^), and the space complexity becomes *O*(*n*^2^) (for representing the distance matrix d). An example of NJ is illustrated in Figure 4.

With a running time of *O*(*n*^3^) on n sequences, NJ is fast and widely used. Moreover, empirical work shows it to be quite accurate, at least for small data sets. St. John et al. [11] even suggest it as a standard against which new phylogeny reconstruction methods should be evaluated. In this paper, we use NJ to refine the unresolved nodes to improve p-ECR.

## Results

In order to test p-ECRNJ, we conducted experiments on real datasets to compare the heuristic ECRML and ECRML+PHYML with four most popular reconstruction methods, including BioNJ [12] (a variant of NJ), PHYML version 2.0.1 [6] (a maximum likelihood algorithm combining hill climbing and NNI moves), RAxML-III[7] (a maximum likelihood algorithm combining hill climbing and SPR moves) and fastDNAml version 1.2.2 [5] (a maximum likelihood algorithm combining the stepwise addition algorithm and SPR moves). ECRML is the heuristic base on p-ECRNJ and hill climbing as shown in Methods. ECRML+PHYML is the heuristic based on the combination of the p-ECRNJ move with NNI, where rounds of NNI and p-ECRNJ are alternated as follows. ECRML is called once each time the PHYML is stuck on a local optimum. If the ECRML is able to improve the tree and get out of the local optimum, the PHYML is applied again until it is trapped in another local optimum, etc. When a pre-defined times is reached or ECRML cannot find an improvement anymore either, the program terminates. The real datasets include the ones used in [7,15], in particular, MouseLemurs, 4DAT, 3DAT, 42, Rbcl55, 101_SC, 132, 150_SC, 150_ARB, 218_RDPII, 250_ARB and 500_ZILLA. Table 1 shows the number of sequences and the number of sites for each dataset.

**Table 1:** Real datasets

	*dataset*	*number of sequences*	*number of sites*
1	MouseLemurs	35	115
2	4DAT	35	452
3	3DAT	39	1116
4	42	42	1167
5	Rbcl55	55	1315
6	101_SC	101	1858
7	132	132	1881
8	150_SC	150	1269
9	150_ARB	150	3188
10	218_RDPII	218	4182
11	250_ARB	250	3638
12	500_ZILLA	500	759

All the programs are run with default options. In addition, the parameter p in ECRML and ECRML+PHYML is set as 4 and iteration times as 20. Computing time is measured on a PC Pentium IV 2.99 GHz running with Windows XP.

Since the BioNJ cannot compute the likelihood values of final trees and there is a difference between all the maximum likelihood algorithms in the way of likelihood computation, all final trees found by BioNJ, PHYML, RAxML and fastDNAml are re-evaluated using ECRML to enable a direct comparison. The main results are shown in Table 2, Table 3 and Table 4. In addition, Stars in Table 2 and Table 4 indicate entries where the algorithm was deemed to be too slow to bother with that test.

**Table 2:** Likelihood values of BioNJ, PHYML, RaxML, fastDNAml and ECRML on different real datasets

	*BioNJ*		*PHYML*		*RAxML*		*fastDNAml*		*ECRML*
		
	likelihood	Δ	likelihood	Δ	likelihood	Δ	likelihood	Δ	
1	-10753	-6902	-5119	-1268	-4959	-1108	-4019	-168	-3851
2	-1082	-1	-1089	-8	-1093	-12	-1082	-1	-1081
3	-2861	-26	-2843	-8	-2842	-7	-2942	-107	-2835
4	-7866	-783	-7250	-167	-7281	-198	-7310	-227	-7083
5	-22552	-299	-22561	-308	-22382	-129	-22603	-350	-22253
6	-67480	-1311	-66695	-526	-66576	-407	-66481	-312	-66169
7	-46930	-3293	-43924	-287	-43641	-4	-43773	-136	-43637
8	-41090	-623	-40520	-53	-40660	-193	-40495	-28	-40467
9	-72423	-1329	-71100	-6	-71159	-65	-71178	-84	-71094
10	-138942	-2035	-137074	-167	-136921	-161	-136998	-91	-136907
11	-120315	-2627	-117869	-181	-118035	-347	****	****	-117688
12	-21917	-588	-22380	-1051	-21879	-550	****	****	-21329

**Table 3:** Likelihood values of various tree building algorithms on different real datasets

	*PHYML*		*ECRML*		*ECRML + PHYML*
		
	likelihood	Δ	likelihood	Δ	
1	-5119	-1275	-3851	-7	-3844
2	-1089	-8	-1081	0	-1081
3	-2843	-13	-2835	-5	-2830
4	-7250	-222	-7083	-55	-7028
5	-22561	-677	-22253	-369	-21884
6	-66695	-511	-66169	15	-66184
7	-43924	-292	-43637	-5	-43632
8	-40520	-80	-40467	-27	-40440
9	-71100	-76	-71094	-18	-71076
10	-137074	-207	-136907	-40	136867
11	-117869	-260	-117688	-79	-117609
12	-22380	-2059	-21329	-1008	-20321

**Table 4:** Computing time(seconds) of various tree building algorithms on different real datasets

*dataset*	*BioNJ*	*PHYML*	*RAxML*	*fastDNAml*	*ECRML*	*ECRML + HYML*
MouseLemurs	3	14	7	187	142	276
4DAT	1	2	2	362	35	55
3DAT	1	7	5	1582	135	205
42	2	31	16	666	449	833
Rbcl55	4	40	89	1586	1340	1733
101_SC	10	155	622	26287	4421	5926
132	8	205	1255	20012	10623	13171
150_sc	24	163	399	26408	7206	9163
150_ARB	24	319	187	54788	25217	28857
218_RDPII	42	429	6779	102388	14236	19897
250_ARB	74	799	1103	****	20788	29804
500_ZILLA	92	2456	29975	****	24528	30016

Table 2 shows the maximum likelihood values of the evolutionary trees reconstructed by BioNJ, PHYML, RAxML, fastDNAml and ECRML on different datasets. Every of the former four algorithms include two columns: the first column lists the maximum likelihood values of the evolutionary trees reconstructed by the algorithm on different datasets; the second column lists the difference of the likelihood value between the algorithm and ECRML on corresponding dataset. A difference that is smaller than 0 means that ECRML can find an evolutionary tree with higher likelihood value than the algorithm on corresponding dataset and vice versa. ECRML only include one column, where lists the likelihood values of ECRML on different datasets. From Table 2, we can see that on every dataset, the values in the second column of BioNJ, PHYML, RAxML and fastDNAml are all smaller than 0. This means that ECRML can find better trees than these four algorithms on all datasets and in further proves that p-ECRNJ has a wider search space.

Table 3 shows the likelihood values of the evolutionary trees reconstructed by PHYML, ECRML and ECRML+ PHYML on different datasets. Similar to Table 2, every of PHYML and ECRML includes two columns: the first column lists the likelihood values of the evolutionary trees reconstructed by the algorithm on different datasets; the second column lists the difference of the likelihood value between the algorithm and ECRML+ PHYML on corresponding dataset. A difference that is smaller than 0 means ECRML + PHYML can find an evolutionary tree with higher likelihood value than the algorithm on corresponding dataset and vice versa. ECRML + PHYML only include one column, where lists the likelihood values of ECRML + PHYML on different datasets. From Table 3, we can see that on every dataset, the values in the second column of PHYML are all smaller than 0. This support that p-ECRNJ can find better trees than other local rearrangements such as NNI and can further efficiently improve them. At the same time, we can also see from Table 3 that there are 10 values smaller than 0, one equal to 0 and one larger than 0 in the second column of ECRML. This means that ECRML + PHYML can often get better trees than ECRML, although they all include a p-ECRNJ search. This is mainly due to that in p-ECRNJ, p edges are randomly deleted; then there is randomicity in p-ECRNJ, which can be eliminated by much iteration. However, when there is no enough iteration, the resulting trees may show some of the defects of starting trees. ECRML is start from a tree reconstructed by NJ and ECRML+PHYML is start from a tree reconstructed by PHYML in each iteration. PHYML can often get better trees than NJ as shown in Table 2. This explains that ECRML+PHYML can often get better trees than ECRML in Table 3.

Table 4 shows the computing time of various tree building algorithms on different real datasets. From table 4, we can see that on every datasets, BioNJ is the fastest. This is in accordance with conclusions that distance based reconstruction methods are often faster than maximum likelihood ones. For the five maximum likelihood methods, fastDNAml, ECRML+PHYML and ECRML are, as a whole, lower than PHYML and RAxML. Currently, PHYML is recognized as the fasted maximum likelihood. The efficiency of PHYML is obtained by simultaneously optimizing tree topology and edge lengths. The efficiency of RAxML comes to a large extent from a very efficient implementation for storing trees and calculating likelihoods. There are no special skills in fastDNAml, ECRML and ECRML+PHYML. Moreover, the computing time of ECRML and ECRML+PHYML is the total of 20 iterations. After each iteration, branches length and likelihood of the current tree is updated; this occupies the majority of the computation time. In terms of coding efficiency, BioNJ, PHYML, RAxML and fastDNAml have been highly brushed up, while the current version of ECRML and ECRML+PHYML is still an experimental program. The computing time for ECRML/ECRML+PHYML is actually the sum of the computing time of the ECRML/ECRML+PHYML subprograms.

At the same time, we can also see from Table 4 that although slower than the two fastest maximum likelihood methods PHYM and RAxML, ECRML and ECRML+PHYML are faster than fastDNAml, especially for large datasets.

## Conclusion

We have proposed the p-ECRNJ move, which can be used as a topological transformation in heuristics on evolutionary tree reconstruction algorithms by itself or can be used to improve local topological transforms. The p-ECRNJ move first randomly select the p edges to contract from the current tree, and then refine the contracted tree to give back a binary tree according to the fast NJ algorithm. Experiments on real datasets show that the p-ECRNJ in limited iterations can find better trees than the best-known maximum likelihood methods so far and can efficiently improve local topological transforms without much time cost. Therefore, the p-ECRNJ is an efficient implementation of p-ECR.

## Methods

In order to make p-ECR efficient, a method p-ECRNJ combining the exhaustiveness of the p-ECR move and the efficiency of NJ is presented in this paper and detailed here. Before p-ECRNJ, several concepts are introduced at first. An evolutionary tree is an unrooted or rooted tree whose leaves have degree one, and all of whose internal nodes have degree at least three. An internal node with degree more than three is called unresolved. A supernode *α *in a tree T is a degree-1 non leaf vertex, denoting some collapsed subtree.

The main idea of the p-ECRNJ is to randomly contract *p *edges from an evolutionary tree T, and the consequent refinement of the unresolved nodes is accomplished by NJ. As show in Figure 4, only one unresolved node Y is successively resolved in NJ. Generally, contracted tree T* in p-ECRNJ contains c (1 ≤ c ≤ p) unresolved nodes. Then, a collapsing procedure is needed before the refinement using NJ. That is, to select an unresolved node to refine and to root the tree at the node, then to collapse every subtree rooted at the node adjacent to the root node into a supernode respectively. This collapsing procedure produces a tree containing only one unresolved node; consequently, the unresolved node is refined according to NJ. The refinement process is continued until there is no unresolved node in T*.

A p-ECRNJ move on an evolutionary tree T is described in detail as follows.

(1) Contraction stage: to randomly select p edges to contract all at once and the unresolved tree T* is resulted;

(2) Refinement stage:

The refinement stage includes the following two steps:

Step 1: Collapsing step:

Select an unresolved node to refine and root T* at the unresolved node, collapse every subtree rooted at the internal node adjacent to the root node into a supernode respectively;

Step 2: NJ step:

1 Build the distance matrix M of the nodes or supernodes in the collapsed tree T*. The distance between two nodes is the distance between the two sequences corresponding the two nodes, respectively. The distance between two supernodes is more sophisticated and computed as follows. Let a and *β *denote two supernodes respectively. It is assumed that a and *β *respectively represents a subtree containing x leaves *α*_*i *_(*i *= 0,..., *x*-1) and a subtree containing y leaves *β*_i _(*i *= 0,..., *y*-1). Then the distance between *α *and *β *is estimated using Eq. (4). The distance between a single node and a supernode is a special case where x = 1 or y = 1.

(4) $$d_{\alpha \beta }=\frac{1}{xy}\left(\sum_{i=0}^{x-1}\sum_{j=0}^{j=y-1}d_{\alpha_{i},\beta_{j}}-\sum_{i=0}^{x-1}d_{\alpha_{i}\alpha_{i+1}}-\sum_{i=0}^{x-1}d_{\beta_{i}\beta_{i+1}}\right)$$

2 According to M, compute matrix Q according to Eq. (1);

3 Select the pair i, j such that min_*i*, *j*_*Q*_*ij *_to agglomerate;

4 Create a new node C which represents the root of the new cluster. Then estimate the length of branches (*C*, *i*) and (*C*, *j*) using Eq. (2);

5 Reduce the distance matrix by replacing the distances relative to *i *and *j *by those between the new node C and any other node k using Eq. (3).

**Figure 5:**
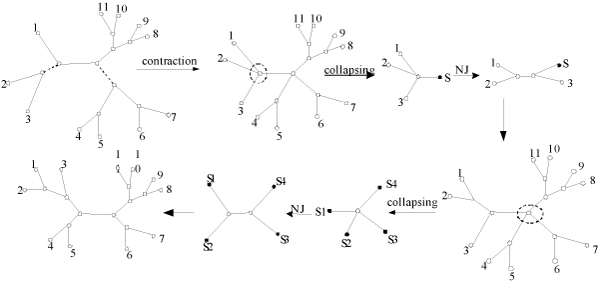
The illustration of a 2-ECRNJ, where the black nodes denote supernodes, white nodes denote leaves or internal nodes, solid and dashed lines represent branches, dashed lines denotes branch to be deleted and the nodes in dashed cycles denote the ones to be refined.

The process of 2, 3, 4 and 5 is repeated until r = 2. The process of collapsing – NJ is repeated until there is no unresolved node in the tree, that is, the number of unresolved nodes *S*_*u *_is 0. The whole p-ECRNJ process is illustrated in Figure 5.

**Figure 6:**
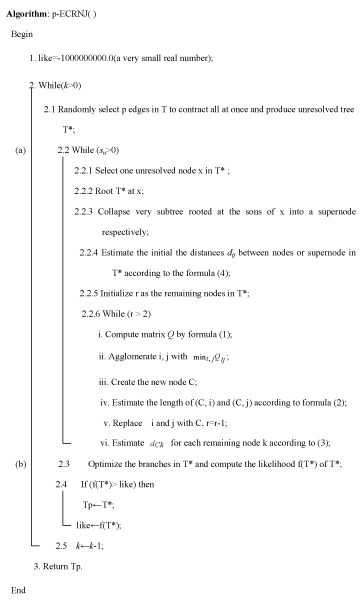
The heuristic ECRML which is based on p-ECRNJ and hill climbing.

Due to that p edges are randomly selected, p-ECRNJ is performed repeatedly a pre-defined times k to perform in actual application instead of considering all $C_{n}^{p}$ ways of selecting p edges to contract. The value of *k *depends on the time allowed, if there is enough time, k can be set to $C_{n}^{p}$. A simple heuristic named ECRML based on p-ECRNJ and hill climbing is shown in Figure 6.

As shown in Figure 6, for every unresolved node, the NJ method is run one time. The time complexity of the NJ method on n sequences is O(n^3^). So, the total time complexity of a p-ECRNJ move is $O\left(\sum_{i=1}^{S_{u}}d_{i}^{3}\right)$, where *S*_*u *_is the number of unresolved nodes and *d*_*i *_is the degree of the unresolved node. As mentioned above, when p edges are deleted from a tree, the location relationship between the deleted edges determines the number of unresolved nodes produced and the degrees of the unresolved nodes. When all the p edges are adjacent, only one unresolved node with degree-(2p) is produced. Then the time complexity of p-ECRNJ is O(8p^3^), that is O(p^3^); when all the p edges disjoin, p unresolved nodes with degree-4 are produced. Then the time complexity of p-ECRNJ is O(4^3 ^p), that is O(p). In other cases, the time complexity is intermediate of the two special cases. Consequently, it takes at most O(p^3^) to refine unresolved nodes in every run of p-ECRNJ(step a). After every p-ECRNJ, it need to optimize the branches and re-compute the likelihood of the current tree(Step b). The time complexity in this step is O(lmn), where l is the iteration times in the optimization of branches, m and n is the number of sites and number of sequences respectively. So, the total time complexity of ECRML is O(k*(p^3^+ lmn)).

In an actual tree search, besides used as a topological transformation operation as shown in Figure 6, the p-ECRNJ move can be combined with a local topological transforms, such as NNI, where rounds of NNI and p-ECRNJ are alternated. For example, ECRML+PHYML in Results is based on the combination of p-ECRNJ and NNI.

## Competing interests

The authors declare that they have no competing interests.

## Authors' contributions

JL conceived, designed and performed the study under the supervision of MG and YL. All authors read and approved the final manuscript.

## References

[B1] Saitou N, Nei M (1987). The neighbor-joining method: a new method for reconstructing phylogenetic Trees. Mol Biol Evol.

[B2] Vincent Ranwez, Olivier Gascuel (2002). Improvement of distance-based phylogenetic methods by a local maximum likelihood approach using triplets. Mol Biol Evol.

[B3] Rosenberg M, Kumar S (2001). Traditional Phylogenetic Reconstruction Methods Reconstruct Shallow and Deep Evolutionary Relationship equally well. Mol Biol Evol.

[B4] Sebastien Roch (2006). A short proof that phylogenetic tree reconstruction by maximum likelihood is hard. IEEE/ACM Transactions on Computational Biology and Bioinformatics.

[B5] Olsen GJ, Matsuda H, Hagstrom R, Overbeek R (1994). fastDNAml: a tool for construction of phylogenetic trees of DNA sequences using maximum likelihood. Comput Appl Biosci.

[B6] Stephane Guindon, Olivier Gascuel (2003). A simple, fast and accurate algorithm to estimate large phylogenies by maximum likelihood. Syst Biol.

[B7] Stamatakis A, Ludwig T, Meier H (2005). RAxML-III: a fast program for maximum likelihood-based inference of large phylogenetic trees. Bioinformatics.

[B8] Lewis P (1998). A Genetic Algorithm for Maximum Likelihood Phylogeny Inference Using Nucleotide Sequence Data. Mol Biol Evol.

[B9] Zwickl DJ (2006). Genetic algorithm approaches for the phylogenetic analysis of large biological sequence datasets under the maximum likelihood criterion, PhD dissertation.

[B10] Ganapathy G, Vijaya Ramachandran, Tandy Warnow (2004). On contract-and-refine transformations between phylogenetic Trees. Proceedings of the Fifteenth ACM-SIAM Symposium on Discrete Algorithms.

[B11] St John K, Warnow T, Moretand B, Vawter L (2003). Performance study of phylogenetic methods: (unweighted) quartet methods and neighbor-joining. Algorithms.

[B12] Gascuel O (1997). BioNJ: an improved version of the NJ algorithm based on a simple model of sequence data. Mol Biol Evol.

[B13] Mary Kuhner K, Felsenstein J (1994). A simulation comparison of phylogeny algorithms under equal and unequal evolutionary rates. Mol Biol Evol.

[B14] Rambaut A, Grassly NC (1997). Seq-Gen: an application for the Monte Carlo Simulation of DNA sequence evolution along phylogenetic trees. Computer Application in Biosciences.

[B15] Wim Hordijk, Olivier Gascuel (2005). Improving the efficiency of SPR moves in phylogenetic tree search methods based on maximum likelihood. Bioinformatics.

